# Re-shifting the ecological baseline for the overexploited Mediterranean red coral

**DOI:** 10.1038/srep42404

**Published:** 2017-02-15

**Authors:** J. Garrabou, E. Sala, C. Linares, J. B. Ledoux, I. Montero-Serra, J. M. Dominici, S. Kipson, N. Teixidó, E. Cebrian, D. K. Kersting, J. G. Harmelin

**Affiliations:** 1 Institut Ciències del Mar (ICM-CSIC), 08003 Barcelona, Spain; 2 Institut Méditerranéen d’Océanologie (MIO), Campus de Luminy, 13288 Marseille Cedex 9, France; 3 National Geographic Society, Washington DC, United States of America; 4 Departament de Biologia Evolutiva, Ecologia i Ciències Ambientals, Universitat de Barcelona, 08028 Barcelona, Spain; 5 CIMAR/CIIMAR, Centro Interdisciplinar de Investigação Marinha e Ambiental, Universidade do Porto, 4050-123 Porto, Portugal; 6 Réserve Naturelle de Scandola, Parc Régional de Corse, Galeria, France; 7 Division of Biology, Faculty of Science, University of Zagreb, 10000 Zagreb, Croatia; 8 Stazione Zoologica Anton Dohrn, 80121 Naples, Italy; 9 Departament de Ciències Ambientals, Facultat de Ciències, Universitat de Girona, 17071, Girona, Spain; 10 Centre d’Estudis Avançats de Blanes-CSIC, 17300, Blanes, Spain; 11 Institut Méditerranéen d’Océanologie (MIO),Station Marine d’Endoume, 13007, Marseille, France

## Abstract

Overexploitation leads to the ecological extinction of many oceanic species. The depletion of historical abundances of large animals, such as whales and sea turtles, is well known. However, the magnitude of the historical overfishing of exploited invertebrates is unclear. The lack of rigorous baseline data limits the implementation of efficient management and conservation plans in the marine realm. The precious Mediterranean red coral *Corallium rubrum* has been intensively exploited since antiquity for its use in jewellery. It shows dramatic signs of overexploitation, with no untouched populations known in shallow waters. Here, we report the discovery of an exceptional red coral population from a previously unexplored shallow underwater cave in Corsica (France) harbouring the largest biomass (by more than 100-fold) reported to date in the Mediterranean. Our findings challenge current assumptions on the pristine state of this emblematic species. Our results suggest that, before intense exploitation, red coral lived in relatively high-density populations with a large proportion of centuries-old colonies, even at very shallow depths. We call for the re-evaluation of the baseline for red coral and question the sustainability of the exploitation of a species that is still common but ecologically (functionally) extinct and in a trajectory of further decline.

Overexploitation in the marine realm has been a major driver of the ecological extinction of many species. This loss dramatically altered the functioning and provisioning of services by marine ecosystems^1^,^2^,^3^. Large marine animals suffered a dramatic decline relative to their abundance in historical records^3^,^4^. This is the case for many species of whales, fishes and sea turtles^4^,^5^. Marine invertebrates have also been the target of fisheries. Some invertebrate species such as abalone, lobsters and sea-urchins have been exploited during centuries even millennia while for others the fisheries just started to expand recently^6^,^7^,^8^. However, for most species, the magnitude of the historical overfishing is unclear. Acquiring precise information of detailed historical baselines is key for setting quantitative targets for guiding conservation goals and management plans.

The Mediterranean red coral *Corallium rubrum* (Linnaeus, 1758) is considered an engineer species (sensu Jones *et al*.^9^) in coralligenous outcrops, which are among the richest but also the most threatened Mediterranean habitats^10^. Red coral have been exploited during millennia. Fisheries peaked during the 1800′ when sail and rowing boats were used to drag a heavy wooden cross with attached nets (St. Andrews Cross) over the bottom to entangle coral colonies. During the last century, the use of motorized vessels allowed a replacement of wooden cross for heavy metal bars (up to 1 tm) (ingegno). The use of this gear caused catastrophic impacts in coralligenous banks^8^,^11^ and its use was banned Mediterranean wide in 1994^12^. Since 1950 s’ scuba diving allowed to exploit colonies dwelling in areas inaccessible by the dredges such as crevices and caves. At present, scuba diving is the only legal way to harvest red coral. This method still threatens the conservation of this species up to deep habitats (up to 140 m depth) when divers used mixed gases. Indeed, overexploitation resulted in unambiguous declines in red coral reported landings during the last decades^12^. With the aim to counteract this negative trend, several management and conservation measures for red coral have been adopted at national and international levels (e.g. Barcelona Convention, FAO GFCM). Overall historical exploitation of red coral challenges the assessment of the pristine state of its populations.

An unexploited population of the precious red coral *Corallium rubrum* was found within the Scandola Marine Reserve in Corsica in 2010 (Fig. 1, Extended data Fig. 1). The population inhabited an unexplored shallow underwater cave-overhang (18 to 27 m in depth; “Cave b” hereafter). The Cave b population exhibited the largest sizes (mean height 9.4 cm and maximum height 28.0 cm) and biomass (3427.4 g m^−2^) reported for similar habitats in the Mediterranean (Supplementary Table 1). The abundance and biomass of large colonies (percent of colonies >10 cm in height or 7 mm in basal diameter) in the Cave b population were 44.3% and 2888.5 g m^−2^, respectively (Fig. 2, Supplementary Table 1 and Extended data Fig. 2). These values are 1 to 3 orders of magnitude greater than those in other populations harbouring large colonies, while in most examined populations, large colonies were simply absent (Fig. 2, and Supplementary Table 1). Therefore, the Cave b population is by far in a more mature state and better preserved than any known populations within the oldest (>35 years old), fully protected marine reserves in the Mediterranean (e.g., populations 3–5 in Fig. 2 and Supplementary Table 2).

Previous research comparing shallow (10–50 m) and deep (50–200 m) habitats in the Western Mediterranean showed that shallow red coral populations are composed of high-density small colonies, and deep populations of large colonies in low-density patches^13^. However, a recent study showed that deep populations in Sardinia (Italy) may also exhibit a wider range of population density^14^. These authors suggested that mature red coral populations must have large colonies in low-density patches regardless of the depth range. This pattern would result from overexploitation and/or self-thinning mechanisms, the latter being analogous to the shift from young to mature trees in forests. However, our results question this assumption because we report the coexistence of a relatively high abundance of small (<3 cm) colonies with large colonies (>10 cm in height) older than 100 years in Cave b (Supplementary Table 1 and Extended data Fig. 2). In addition, our findings show no significant relationship between population density (colonies per square metre) and size (mean colony biomass per square metre) in either protected (R^2^ = 0.123, p > 0.1) or unprotected (R^2^ = 0.086, p > 0.3) populations thriving in shallow habitats (Fig. 3). Accordingly, the current working hypothesis stating that red coral population recovery after exploitation occurs through population thinning as colonies grow in size is not supported by our study (Fig. 3). Finally, we have to bear in mind that, besides intraspecific processes, community-level interactions and environmental drivers may also shape the relationship between density and size of colonies in red coral populations.

The magnitude of loss of large red coral colonies due to overexploitation throughout its geographical range (nearly the entire Mediterranean Sea and the neighbouring Atlantic coasts, see the Methods section) is comparable to the mass deforestation at a continental scale (as evidenced in the Amazonian forest^15^). Individual colonies of *C. rubrum* up to 50 cm tall and hundreds of years old found in museums and private collections are now virtually absent in the first 100 m in depth. The sizes of the colonies found in Cave b are similar to the size of those found in the past in shallow water habitats in the Mediterranean. The current exploitation of deep habitats is further accelerating the loss of the last mature populations beyond 100 m in depth^16^. Given the absence of large colonies in dense populations, red coral cannot exert its former ecological role as a habitat-forming species^17^; thus, red coral should be considered ecologically extinct. The effects of overexploitation affect not only red coral but also many other species in its habitat^18^,^19^. The magnitude of the human-caused loss of red coral in the Mediterranean provides strong evidence of the shifting-baseline syndrome (“changing human perceptions of biological systems due to loss of experience about past conditions^20^,^21^”) for this species in shallow habitats. Even populations that have been fully protected for more than 3 decades only show initial signs of recovery (Fig. 2). Therefore, we suggest that the Cave b population may be the rule, instead of the exception, and should be used to reset the baseline for Mediterranean red coral populations.

Despite thousands of years of exploitation, red coral is still commonly observed in the Mediterranean. However, its populations are dominated by small colonies. This fact challenges our perception of its conservation status. The persistence of overexploited populations is possible due mainly to the capacity of red coral colonies to regenerate when they are affected by fishing. In these populations, the high survival and re-growth capacity of partially broken colonies (fished and still attached to the substratum) facilitate the persistence of these colonies^22^, thereby perpetuating the overexploited state. However, when harvesting practices cause whole-colony mortality in the populations, the loss in abundance (both in density and size) may drive most red coral populations to functional collapses and ultimately to local population extinctions. This likely occurs because self-recruitment has been identified as a central process for population persistence^16^ (except in a few high-density populations), along with the increase in mortality sources (such as seawater warming^23^,^24^,^25^). Overall, red coral populations are characterized by slow growth and low natural mortality rates^26^, resulting in an apparent stability when in fact they are pushed in declining trajectories^27^. In the context of local and global pressures impacting red coral populations, our discovery provides sound basis for an urgent re-evaluation and re-definition of the current management plans and conservation goals for this emblematic Mediterranean species. New measures should establish much larger fishing minimum legal colony sizes, fishing moratorium as well as the implementation a network of well enforced no take protected areas around the Mediterranean to ensure the full recovery to mature states of red coral populations.

## Methods

### The species

The Mediterranean red coral *Corallium rubrum* (Linnaeus, 1758) is considered an engineer species (sensu Jones *et al*.^9^) in coralligenous outcrops, which are among the richest but also most threatened Mediterranean habitats^10^. *C. rubrum* displays a fragmented distribution across the entire Mediterranean; however, the most abundant populations are found in the western Mediterranean basin and the neighbouring Atlantic coasts from south Portugal and northern Morocco^16^,^28^. *C. rubrum* dwells in heterogeneous habitats, as illustrated by its bathymetric distribution, ranging from 5 to 800 m in depth^29^. Red coral shallow populations are commonly found along crevices, overhangs and cave entrances while in deeper water habitats colonies can be found either on horizontal surfaces and in overhangs^30^,^31^,^32^. This species is a sessile, aposymbiotic, long-lived cnidarian that exhibits slow population dynamics^26^,^33^ and late sexual maturity (at approximately 10 years of age and small size about 25 and 3.6 mm in height and diameter respectively^34^). Red coral populations are genetically structured at a scale of tens of metres^21^,^22^,^23^,^35^,^36^,^37^, in accordance with the restricted effective dispersal of this species^38^ Red coral displays very slow growth rates (approximately 0.24–0.26 mm year^−1^ in diameter) in both shallow and deep waters and may have a life span of several decades and potentially even centuries^26^,^39^,^40^. Furthermore, long-term studies show low recruitment rates in most analysed populations (<0.25 recruits dm^−2^ year^−1^)^26^,^41^. The low recruitment and slow growth rates displayed by this species do not seem to counterbalance the loss of density caused by escalating human- and climate-induced mortalities that have affected red coral populations in recent decades^25^,^41^,^42^. As a result of overexploitation, red coral populations are currently dominated by small-sized colonies in regards to the historical sizes^26^.

### Study site

Cave b (42.3802° (N) 8.54635° (E)) is situated in the Reserve Naturelle de Scandola in the Parc Naturel Regional de Corse (Corsica, France) in the Northwestern Mediterranean Sea. The Réserve Naturelle de Scandola was established in 1975 and was acknowledged as a UNESCO world heritage site in 1980. This well enforced reserve is characterized by an exceptional level of marine biodiversity and serves as a worldwide reference for scientist and manager communities. The zonation in the reserve allows for different levels of protection. Cave b is situated within the no-take zone, featuring the highest level of protection and where fishing, diving and all other activities are strictly prohibited. Cave b is a dim-light, large overhang cave situated between 18 m and 27 cm in depth with an average width approximately of 30 m, resulting in an estimated total area covered by the Cave b red coral population of approximately 300 m^2^.

### Cave b population structure

Data on the size structure and density of the Cave b population were obtained from 23 quadrats (20 × 20 cm) randomly placed between 22 and 24 m in depth. Within each quadrat, we counted and measured the maximum height of all of the colonies that were present using a ruler (0.5 cm accuracy). A total of 185 colonies were measured. The data on the colony height were used to quantify the mean, standard deviation and range of maximum height, whereas the size structure was obtained by pooling colonies into 1 cm size classes between 0 and 28 cm (Extended data Fig. 2). The age of the largest colonies from the Cave b is estimated above 100 years old. This statement was based on the assumption that growth rates in the Cave b population are similar to those found in Palazzu population (less than 1 km away from the Cave b)^43^. For this population aging estimations resulted in colonies up to 200 years old^43^. Bearing in mind that the Palazzu population size structure was less mature than in the Cave b population (i.e. lower mean size and maximum height) (Extended Data Table 1 and Linares *et al*.^43^), we contend that age estimates in the Cave b are not overestimated. The population density was estimated by averaging the number of colonies found in each quadrat (Supplementary Table 1).

### Populations size structure and density data

To compare the Cave b population structure with that of other red coral populations, we compiled data on colony size (height and/or diameter), size structure and density from the literature^8^,^44^,^45^. Whenever data on size structure were not available from the literature, we requested the original datasets directly from the authors. Overall we compiled population structure data for 34 populations covering the Northwestern Mediterranean Basin (Supplementary Table 2). The density values were standardized to colonies per square metre for comparative and biomass estimation purposes (see below) (Supplementary Data Table 1). Because the Cave b population dwells in shallow depths, we limited our analysis to populations developing in a depth range between 10 and 50 m in depth, except for three populations that dwell between 50 and 80 m in depth (Supplementary Table 2).

### Population biomass assessment

To obtain the population biomass, we first estimated the mean weight per colony in each size class. The colony biomass was estimated by applying a height-weight power equation [Weight (g) = 0.1535 (Height, cm)^1,9732^ (R^2^ = 0.863, p < 0.001)] and diameter-weight power equation [Weight (g) = 0.0152 (Diameter, mm)^2,9943^ (R^2^ = 0.5043, p < 0.001)]. The equations were obtained in this study from a non-linear regression analysis between height and weight data and diameter–weight data from 300 dead red coral colonies ranging from 2 to 21 cm in height and 2.6 to 14.4 mm in diameter collected in previous studies and from different poaching events in the Northwestern Mediterranean by the authors^41^,^46^. Second, we combined density and size structure data to obtain the number of colonies in each size class per square metre for each population. Finally, we multiplied the number of colonies per size class by the mean weight of colonies in each size class. Summing the biomass obtained per size class, we obtained either the total population biomass per square metre or the biomass corresponding only to large colonies (height >10 cm or diameter >7 mm) (see below). In total, we estimated the biomass in 22 populations corresponding to populations for which original datasets on height (19) and/or diameter (3) were available (Supplementary Table 1). For the other 12 populations^47^, we reported total biomass data directly estimated by the authors. However, in these populations, we could not assess the biomass corresponding to the large colonies because data on size structure were not available for the analysis.

### Assessment of the conservation status of the Cave b population

**Figure 2:**
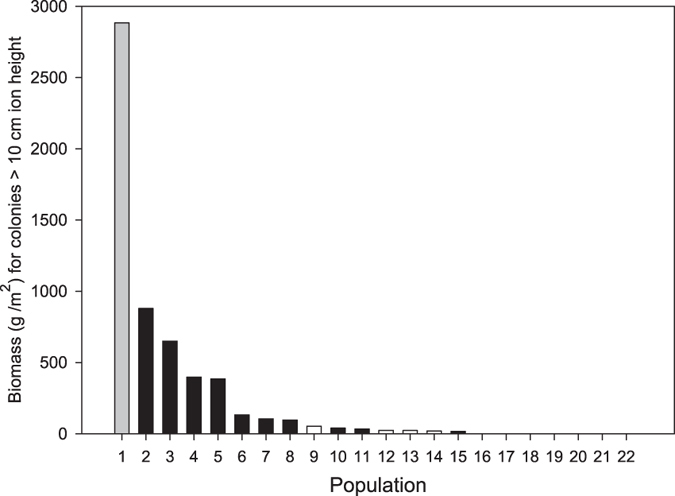
Biomass (g/m^2^; see Methods for computation details) accounting for colonies larger than 10 cm in height or 0.7 cm in diameter. The value of the red coral population from Cave b (Population 1 in grey) is compared to the values of 22 populations reported to date in the literature (Supplementary Table 1). Populations from marine protected areas are shown in black; unprotected populations are shown in white.

Because the major disturbance to red coral populations is harvesting, assessing the descriptors of abundance of large colonies provides reliable insight into the conservation status of populations. Large colonies provide the architectural complexity necessary for natural population and community functioning. It has been suggested the conservation status of *C. rubrum* populations can be well approximated by quantifying the proportion of colonies greater than >10 cm in height and >7 mm in diameter^43^ (Supplementary Table 1). Here, we computed the biomass provided by large colonies to each population (Fig. 2 and Supplementary Table 1). Finally, to examine the potential recovery trajectories in red coral populations through the occurrence of self-thinning, we plotted the mean biomass per colony vs. density. We separately analysed populations from protected and unprotected areas because the population trajectories in these contrasted management schemes are expected to differ.

## Additional Information

**How to cite this article**: Garrabou, J. *et al*. Re-shifting the ecological baseline for the overexploited Mediterranean red coral. *Sci. Rep.* **7**, 42404; doi: 10.1038/srep42404 (2017).

**Publisher's note:** Springer Nature remains neutral with regard to jurisdictional claims in published maps and institutional affiliations.

## Supplementary Material

Supplementary material Garrabou et al

## Figures and Tables

**Figure 1:**
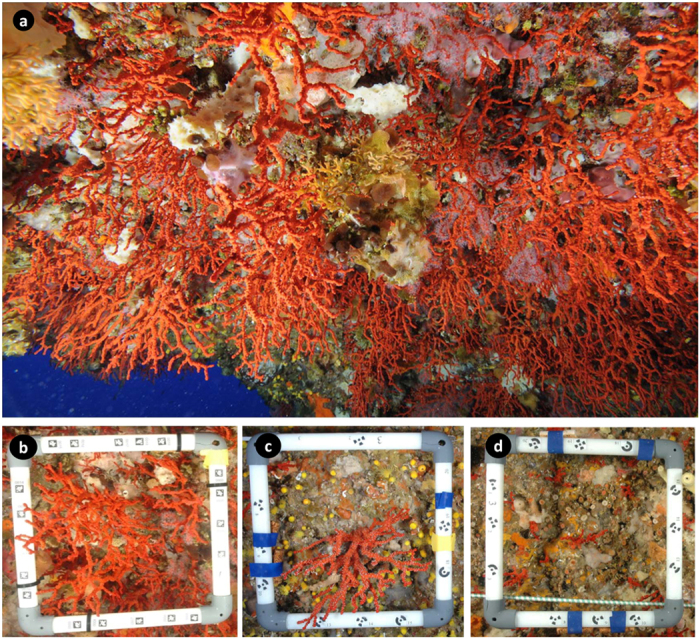
The precious Mediterranean red coral, *Corallium rubrum*: (**a**) Overview of the red coral population discovered between 18 and 27 m at Cave b in the Scandola Marine Reserve (Parc Naturel Régional de Corse, France); (**b–d**) Different states of shallow red coral populations scaled with a 20 × 20 cm quadrat; (**b**) the population from Cave b consisting of a high density (201 colonies m^−2^) of large colonies (9.4 cm in height). (**c**: a Typical population from a marine protected area (Scandola) consisting of a low density (70 colonies m^−2^) of large colonies (6.7 cm in height); (**d**) a standard population from an unprotected area (Provence, France) consisting of a relatively high density (466 colonies m^−2^) of small colonies (2.3 cm in height).

**Figure 3:**
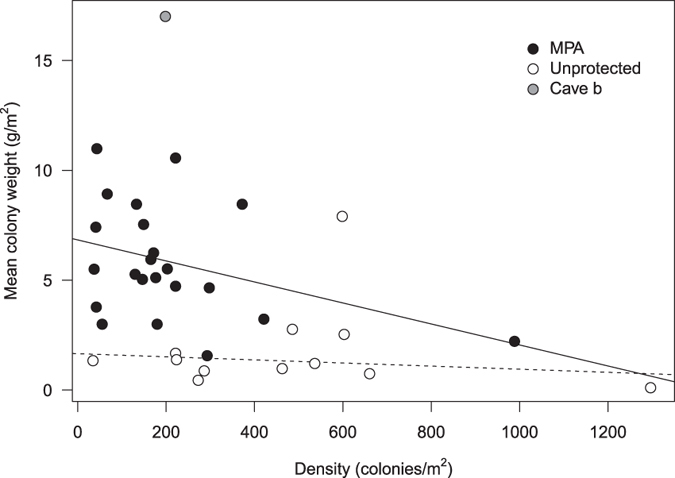
Mean colony weight (biomass (g)/number of colonies) as a function of density (colonies m^−2^) for the 34 populations analysed: Cave b (grey dot); unprotected populations (white dots); and protected populations (black dots) (Supplementary Tables 1 and 2).
