# Supplementary material for: Re-shifting the ecological baseline for the overexploited Mediterranean red coral
Source: Sci Rep. 2017 Feb 15;7:42404. doi: 10.1038/srep42404 (PMC5309836; doi:10.1038/srep42404)
Supplement: Supplementary material Garrabou et al [file srep42404-s1.pdf]

Re-shifting the ecological baseline for overexploited Mediterranean red coral

Authors: Garrabou J[1,2], Sala E[3], Linares C[4], Ledoux JB[1,5], Montero I[4], Dominici JM[6], Kipson S[7], Teixidó N[1,8], Cebrian E[9,10], Kersting DK[4], Harmelin JG[2]

Extended data Figures

Extended data Figure 1. Map of the Northwestern Mediterranean with the geographic position of the Cave b population (red dot) and the protected (black dots) and unprotected (white dots) locations of red coral populations that were included in the analysis (see Supplementary Table 1 and 2 providing the main features of populations). Maps was generated with marmap package v. 0.9.5 in R environment v. 3.3.0 (http://www.R-project.org/)[1]

Extended data Figure 2. Size structure height (cm) of the Cave b population obtained from the measurements of the maximum height of the colonies found in 23 (20 x 20 cm) quadrats.

Supplementary Tables

Supplementary Table 1. This file contains information on the population structure (density, size and biomass) for the Cave b population and for protected and unprotected populations reported in the literature.

Supplementary Table 2. This file contains information on the geographic position, depth range, sampling year, habitat, level and year of protection for the populations considered in this study.

[1] References to be included in the references list if needed

R Core Team (2016). R: A language and environment for statistical computing. R Foundation for Statistical Computing, Vienna, Austria. URL http://www.R-project.org/
Pante E, SimonBouhet B (2013) marmap: a package for importing, plotting and analyzing bathymetric and topographic data in R. PLoS ONE 8(9): e73051. doi: 10.1371/journal.pone.0073051

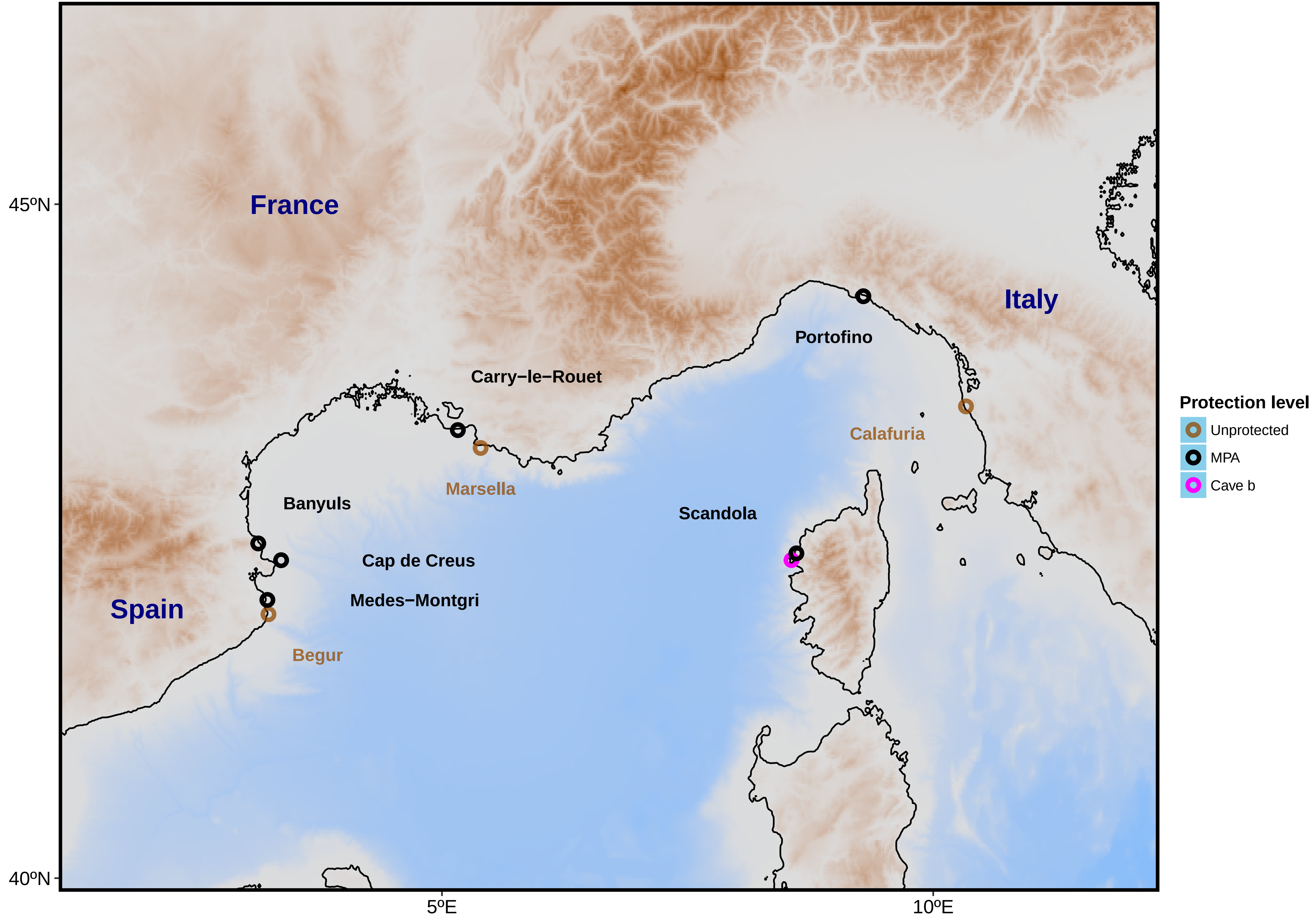
France
Italy
Spain
Portofino
Carry−le−Rouet
Calafuria
Marsella
Banyuls
Scandola
Cap de Creus
Medes−Montgri
Begur
45ºN
40ºN
5ºE
10ºE
Protection level
Unprotected
MPA
Cave b

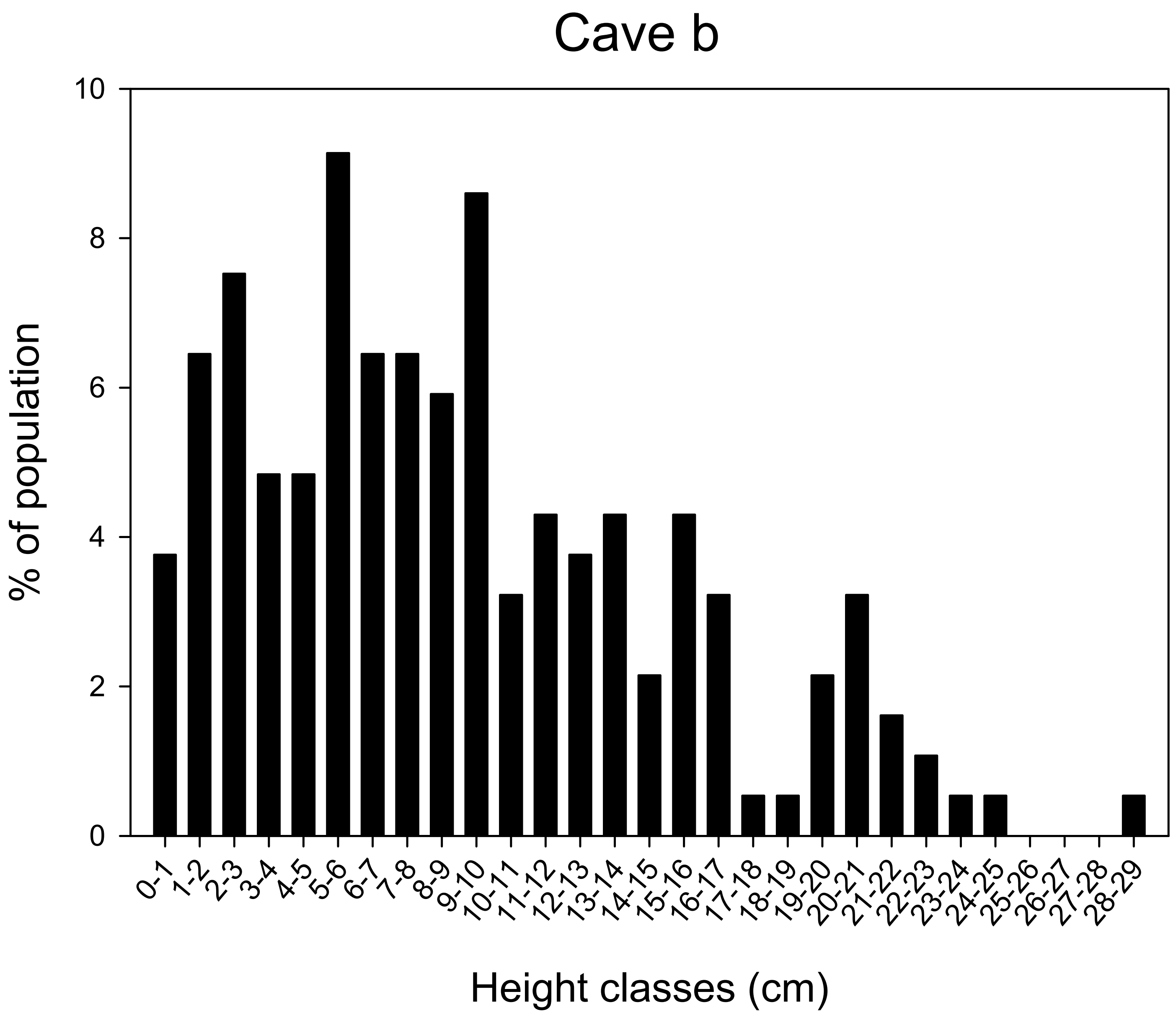

Cave b
% of population
0
2
4
6
8
10
0-1
1-2
2-3
3-4
4-5
5-6
6-7
7-8
8-9
9-10
10-11
11-12
12-13
13-14
14-15
15-16
16-17
17-18
18-19
19-20
20-21
21-22
22-23
23-24
24-25
25-26
26-27
27-28
28-29
Height classes (cm)

ID	Population	N (col)	Density (col m^{-2})	Biomass (g m^{-2})	Height (cm) Mean	SD	Min-Max	Diameter (mm) Mean	SD	Min-Max	Large colonies % > 10 cm height	% > 7 mm diameter	Biomass (g m^{-2})	Reference
1	Cave b	185	201,0	3427,4	9,4	5,8	0,5-28,0	-	-	-	44,3	-	2883,5	This study
2	Portofino	143	992,5	2251,8	-	-	-	4,4	1,7	na-14,5	-	8,7	880,0	30
3	Banyuls	109	137,0	1164,1	6,7	3,8	0,9-16,6	-	-	-	20,0	-	650,6	33
4	Carry-le-Rouet	89	47,0	523,4	7,4	4,7	0,6-15,3	-	-	-	38,0	-	397,4	33
5	Palazzu	166	70,0	629,2	6,7	4,2	0,4-16,9	8,9	5,8	na-29,7	24,9	54,2	385,1	33
6	Cap de Creus	477	297,5	477,3	-	-	-	4,1	1,2	na - 11,5	-	9,0	133,2	30
7	Medes shallow	819	184,4	554,5	4,2	2,6	0 - 19,0	6,9	2,4	2,0 - 30,0	2,6	48,4	105,2	29
8	Tres Frares	NA	45,0	335,2	5,7	2,9	1,4 - 13,5	7,0	4,0	3,0 - 21,0	11,3	43,7	96,9	5
9	Figuier Exterior	331	606,6	1555,0	3,8	1,7	0,2-10,0	-	-	-	0,6	-	53,0	12
10	Pedres Barra Massin	NA	42,0	232,5	5,5	2,6	1,9 - 15,5	9,0	4,0	4 ,0- 22,0	3,7	58,5	39,8	5
11	Medes deep	819	58,9	177,1	4,2	2,6	0 - 19,0	6,9	2,4	2,0 - 30,0	2,6	48,4	33,6	29
12	Moyade Exterior	137	227,9	326,6	2,7	1,6	0,3-10,0	-	-	-	0,7	-	24,1	12
13	Calafuria	4408	1300,0	201,5	-	-	-	1,3	1,0	0,0-8,0	-	0,3	23,2	31
14	Moyade Interior	196	275,7	138,7	1,6	0,9	0,2-10,0	-	-	-	0,5	-	19,9	12
15	Montgrí	695	224,5	383,7	2,6	1,4	0 - 10,0	4,9	2,1	2 ,0- 30,0	0,1	15,5	16,3	29
16	Begur	1051	38,4	53,0	3,0	1,7	0 - 10,0	4,9	1,5	2,0 - 20,0	0,1	14,9	0,6	29
17	Plane Exterior	266	466,9	476,0	2,3	1,2	0,2-7,0	-	-	-	0,0	-	0,0	12
18	Plane Interior	279	489,0	1382,2	3,9	0,2	0,3-9,5	-	-	-	0,0	-	0,0	12
19	Figuier Interior	166	290,4	271,7	2,2	1,3	0,1-6,0	-	-	-	0,0	-	0,0	12
20	Grotte Pérès	175	665,0	528,9	2,0	1,1	0,5-5,2	-	-	-	0,0	-	0,0	33
21	Maire	150	540,0	683,4	2,6	1,2	0,6-6,4	-	-	-	0,0	-	0,0	33
22	Massa d'oros	NA	45,0	170,7	4,9	1,7	1,8 - 7,9	6,0	2,0	3,0 - 14,0	0,0	21,9	0,0	5
23	Isuela Sud	NA	225,0	2390,4	6,3	0,3	-	8,2	0,7	-	-	-	-	32
24	Cala Oro Est	NA	375,6	3172,5	8,6	1,3	-	12,1	1,8	-	-	-	-	32
25	Torreta	NA	150,0	772,5	6,9	0,8	-	9,5	0,1	-	-	-	-	32
26	Dragone	NA	175,0	1105,0	6	0,4	-	7,2	0,9	-	-	-	-	32
27	Targa Gonzatti	NA	168,3	1005,0	6,9	0,4	-	7,0	0,9	-	-	-	-	32
28	Diamante	NA	300,0	1402,5	5,8	0,6	-	10,1	1,3	-	-	-	-	32
29	Altare	NA	205,0	1040,0	5	0,5	-	7,8	0,8	-	-	-	-	32
30	Punta Vessinaro	NA	135,0	816,6	5,1	0,4	-	6,2	0,8	-	-	-	-	32
31	Casa del Sindaco	NA	153,7	1192,5	6,1	0,3	-	12,1	1,7	-	-	-	-	32
32	San Giorgio	NA	181,2	826,2	6,2	0,4	-	9,6	1,2	-	-	-	-	32
33	San Giorgio Est	NA	225,6	225,6	4,6	0,3	-	8,4	1,1	-	-	-	-	32
34	Faro cape	NA	425,0	1415,0	4,6	0,5	-	6,9	0,5	-	-	-	-	32

ID	Population	Location	Area	Country	Latitude	Longitude	Depth range (m)	Sampling year	Habitat	Protection level	Year of protection
1	Cave b	Scandola	W Corsica	France	42.3597	8.5575	22-24	2011	Cave-overhang	MPA	1975
2	Portofino	Portofino	Ligurian Sea	Italy	44.3030	9.2138	30-35	2009	NA	MPA	1999
3	Banyuls	Banyuls	Gulf of Lions	France	42.4833	3.1305	23–25	2005	Overhang	MPA	1974
4	Carry-le-Rouet	Carry-le-Rouet	Gulf of Lions	France	43.3241	5.1619	24-25	2005	Overhang	MPA	1983
5	Palazzu	Scandola	Corsica	France	42.4097	8.6075	19-22	2003	Cave	MPA	1975
6	Cap de Creus	Cap de Creus	Catalan Sea	Spain	42.4868	3.5030	30-35	2009	NA	MPA	1998
7	Medes shallow	Medes-Montgri	Catalan Sea	Spain	42.0467	3.2273	15-49	2002-03	NA	MPA	1983
8	Tres Frares	Cap de Creus	Catalan Sea	Spain	42.3393	3.3019	70-80	2003	Boulders	MPA	1998
9	Figuier Exterior	Marsella	Provence	France	43.2051	5.4469	17	1999-2000	Overhang	Unprotected*	
10	Pedres Barra Massina	Cap de Creus	Catalan Sea	Spain	42.2937	3.3142	60-73	2002/03	Boulders	MPA	1998
11	Medes deep	Medes-Montgri	Catalan Sea	Spain	42.0474	3.2284	15-49	2002-03	Overhang	MPA	1983
12	Moyade Exterior	Marsella	Provence	France	43.1770	5.3721	20-25	1999-2000	Overhang	Unprotected*	
13	Calafuria	Calafuria	Ligurian Sea	Italy	43.5000	10.3333	25-40	1992-97	Steep wall	Unprotected	
14	Moyade Interior	Marsella	Marseille	France	43.1772	5.3723	20-25	1999-2000	Cave	Unprotected*	
15	Montgrí	Medes-Montgri	Catalan Sea	Spain	42.0989	3.2142	13-32	2002-03	NA	MPA	1992
16	Begur	Begur	Catalan Sea	Spain	41.9581	3.2346	18-32	2002-03	NA	Unprotected	
17	Plane Exterior	Marsella	Provence	France	43.1865	5.3915	13-15	1999-2000	Cave	Unprotected*	
18	Plane Interior	Marsella	Provence	France	43.1866	5.3912	13-15	1999-2000	Cave	Unprotected*	
19	Figuier Interior	Marsella	Provence	France	43.2052	5.4469	10-15	1999-2000	Cave	Unprotected*	
20	Grotte Pérès	Marsella	Provence	France	43.1866	5.3913	13-15	2003	Cave	Unprotected*	
21	Maire	Marsella	Provence	France	43.2090	5.3372	15-18	2004	Tunel	Unprotected*	
22	Massa d'oros	Cap de Creus	Catalan Sea	Spain	42.3155	3.3337	50-80	2002/03	Steep wall	MPA	1998
23	Isuela Sud	Portofino	Ligurian Sea	Italy	44.3814	9.3194	25-40	2012	Steep wall	MPA	1999
24	Cala Oro Est	Portofino	Ligurian Sea	Italy	44.4972	9.1806	25-40	2012	Steep wall	MPA	1999
25	Torreta	Portofino	Ligurian Sea	Italy	44.5094	9.1822	25-40	2012	Steep wall	MPA	1999
26	Dragone	Portofino	Ligurian Sea	Italy	44.4936	9.3181	25-40	2012	Steep wall	MPA	1999
27	Targa Gonzatti	Portofino	Ligurian Sea	Italy	44.4400	9.3836	25-40	2012	Steep wall	MPA	1999
28	Diamante	Portofino	Ligurian Sea	Italy	44.4353	9.2144	25-40	2012	Steep wall	MPA	1999
29	Altare	Portofino	Ligurian Sea	Italy	44.3917	9.4067	25-40	2012	Steep wall	MPA	1999
30	Punta Vessinaro	Portofino	Ligurian Sea	Italy	44.3703	9.4336	25-40	2012	Steep wall	MPA	1999
31	Casa del Sindaco	Portofino	Ligurian Sea	Italy	44.3494	9.2497	25-40	2012	Steep wall	MPA	1999
32	San Giorgio	Portofino	Ligurian Sea	Italy	44.3103	9.3853	25-40	2012	Steep wall	MPA	1999
33	San Giorgio Est	Portofino	Ligurian Sea	Italy	44.3972	9.2181	25-40	2012	Steep wall	MPA	1999
34	Faro cape	Portofino	Ligurian Sea	Italy	44.5461	9.2550	25-40	2012	Steep wall	MPA	1999

* This area has been declared protected in 2012

Supplementary table 2
